# Innate Immunity Provides Biomarkers of Health for Teleosts Exposed to Nanoparticles

**DOI:** 10.3389/fimmu.2018.03074

**Published:** 2019-01-09

**Authors:** Débora Torrealba, Juan A. More-Bayona, Jeremy Wakaruk, Daniel R. Barreda

**Affiliations:** ^1^Immunology and Animal Health Laboratory, Department of Biological Sciences, University of Alberta, Edmonton, AB, Canada; ^2^Department of Agricultural, Food and Nutritional Science, University of Alberta, Edmonton, AB, Canada

**Keywords:** innate immunity system, nanoparticles, teleosts, oxidative stress, leukocytes

## Abstract

In recent years, the unique properties of nanoparticles have fostered novel applications in various fields such as biology, pharmaceuticals, agriculture, and others. Unfortunately, their rapid integration into daily life has also led to environmental concerns due to uncontrolled release of nanoparticles into the aquatic environment. Despite increasing awareness of nanoparticle bioaccumulation in the aquatic environment, much remains to be learned about their impact on aquatic organisms and how to best monitor these effects. Herein, we provide the first review of innate immunity as an emerging tool to assess the health of fish following nanoparticle exposure. Fish are widely used as sentinels for aquatic ecosystem pollution and innate immune parameters offer sensitive and reliable tools that can be harnessed for evaluation of contamination events. The most frequent biomarkers highlighted in literature to date include, but are not limited to, parameters associated with leukocyte dynamics, oxidative stress, and cytokine production. Taken together, innate immunity offers finite and sensitive biomarkers for assessment of the impact of nanoparticles on fish health.

## Introduction

Nanoparticles (NPs) are an emerging technology, currently being applied in various fields including medicine, cosmetics, electronics, space science, chemical manufacturing, cellular and molecular biology, agricultural and animal science (1–4). Nanomaterials are structures of 1–1,000 nm in size; however, stricter definitions restrict them to those in the 1–100 nm range (National Nanotechnology Initiative). NPs have been developed in many different inorganic and organic forms but most current NPs are classified into four material-based categories: (i) carbon based NPs such as fullerenes and carbon nanotubes; (ii) inorganic-based NPs including metal (e.g., gold NPs, silver NPs) and metal oxide NPs (e.g., titanium dioxide NPs, zinc oxide NPs); (iii) organic-based NPs such as dendrimers, liposomes, polymers; and (iv) composite-based NPs: combinations of different material-based NPs (1, 2).

The rising use of NPs in modern technologies has led to unregulated release and accumulation into the aquatic environment, contributing to further pollution (5, 6). For example, aquatic release of titanium dioxide NP (TiO_2_) has been estimated at 17 % of their total production per year (7). Based on a production scale of 88 kt/year, this translates into a significant aquatic release of 15.6 kt/year. Releases for other nanomaterials have been recently summarized (7). NPs enter the aquatic environment mainly through wastewater and effluents from industrial sources, as well as via atmospheric deposition, leaching from soil, accidental spillages, and agricultural drainage water (1, 5). Naturally-occurring NPs also enter aquatic environments through waterways across several landscapes (8). Despite our growing knowledge about NP bioaccumulation within aquatic environments, little is known about the detrimental effects of nanoparticles on animal and human health. Various features such as size, chemical makeup, biodegradability, and the physicochemical environment all affect the extent to which NPs are contributors of biotoxicity (5, 9).

Fish are widely used as sentinels for aquatic ecosystem pollution stemming from chemical exposure and are the preferred model for development of chemical testing guidelines (10, 11). Among others, their practical relevance are based on: (i) wide distribution in aquatic environments; (ii) high ecological relevance due to position within food web structure, nutrient cycling and energy transfer; and (iii) expanding models and tools that researchers can use (12). In addition, testing on native species under natural housing conditions (e.g., *Oncorhynchus mykiss* to test freshwater pollution and aquatic toxicity in Canadian cool waters) adds relevance to these datasets (13). Consequently, fish species have now become a preferred model to study NP toxicity (14–16). A summary for recent applications of innate immune parameters as biomarkers of impact on animal health is provided in Tables 1, 2.

**Table 1:** Innate immune defenses used as biomarkers to assess fish health post exposure to nanoparticles.

**Innate immune defenses**	**Nanoparticles**	**References**
**EXTERNAL BARRIERS**		
Mucus		
	Gold (Au)	(17)
	Silver (Ag)	(18)
Gills		
	Aluminum oxide (Al_2_O_3_)	(19)
	Copper (Cu)	(20, 21)
	Copper oxide (CuO)	(22–24)
	Graphene oxide (GO)	(25)
	Gold (Au)	(26)
	Iron oxide (Fe_2_O_3_)	(27)
	Multi walled carbon nanotubes (MWCNTs)	(28)
	Silica coated iron oxide (Fe_3_O_4_-SiO_2_)	(29)
	Silver (Ag)	(21, 30–36)
	Silver nitrate (AgNO_3_)	(34)
	Single wallet carbon nanotubes (SWCNTs)	(37, 38)
	Titanium dioxide (TiO_2_)	(39)
	Zinc oxide (ZnO)	(19, 40, 41)
**CELLULAR RESPONSE**		
Leukocytes		
**Neutrophils**		
	Cerium oxide (CeO_2_)	(42)
	Copper (Cu)	(43)
	Gold (Au)	(43)
	Hydroxylated fullerenes (C_60_(OH)_24_)	(44)
	Iron oxide (Fe_2_O_3_)	(42)
	Polycarbonate	(45)
	Polystyrene	(45)
	Silica (Si)	(46, 47)
	Silver (Ag)	(43)
	Titanium dioxide (TiO_2_)	(42, 48)
	Zinc oxide (ZnO)	(42)
Macrophages		
	Hydroxylated fullerenes (C_60_(OH)_24_)	(44)
	Iron oxide (Fe_2_O_3_)	(49)
	Silica (Si)	(50)
	Titanium dioxide (TiO_2_)	(48)
Lymphocytes		
	Cadmium sulfate quantum dots (CdSQDs)	(51)
	Cadmiun telluride quantum dots (Cd-TeQDs)	(51)
	Copper (Cu)	(43)
	Copper oxide (CuO)	(52)
	Gold (Au)	(43)
	Hydroxylated fullerenes (C_60_(OH)_24_)	(44)
	Silver (Ag)	(43)
	Titanium dioxide (TiO_2_)	(48, 53)
	Cadmium sulfate quantum dots (CdSQDs)	(51, 54)
	Cadmiun telluride quantum dots (Cd-TeQDs)	(51, 54, 55)
	Copper (Cu)	(43)
	Cobalt ferrite (CoFe_2_O_4_)	(56)
	Gold (Au)	(43, 57)
	Poly (acrylic acid)	(58)
	Polycarbonate	(45)
	Polystyrene	(45, 57)
	Poly (lactic-co-glycolic acid) (PLGA)	(57)
	Silica coated iron oxide (Fe_3_O_4_-SiO_2_)	(29, 59)
	Silver (Ag)	(43, 60, 61)
	Titanium dioxide (TiO_2_)	(62, 63)
	Zinc oxide (ZnO)	(40)
**OXIDATIVE STRESS**		
**ROS**		
	Cerium oxide (CeO_2_)	(42)
	Cobalt ferrite (CoFe_2_O_4_)	(56)
	Cobalt oxide (Co_3_O_4_)	(64)
	Copper (Cu)	(43, 65)
	Copper oxide (CuO)	(22, 64, 66, 67)
	Copper sulfate (CuSO_4_)	(65)
	Fullerene C_60_	(68)
	Graphene oxide (GO)	(25)
	Gold (Au)	(43, 69)
	Hydroxylated fullerenes (C_60_(OH)_24_)	(44)
	Iron oxide (Fe_3_O_4_)	(70)
	Iron oxide (Fe_2_O_3_)	(27, 42)
	Polyamidoamine (PAMAM)	(71)
	Polycarbonate	(45)
	Polystyrene	(45)
	Silica (Si)	(46, 47)
	Silica coated iron oxide (Fe_3_O_4_-SiO_2_)	(29)
	Silver (Ag)	(43, 60, 72)
	Single wallet carbon nanotubes (SWCNTs)	(37)
	Titanium dioxide (TiO_2_)	(39, 42, 48, 64)
	Zinc oxide (ZnO)	(40–42, 64, 73, 74)
Nitric oxide (NO)		
	Cerium oxide (CeO_2_)	(75)
	Copper oxide (CuO)	(66, 75)
**ANTIOXIDANT DEFENSES**		
Superoxide dismutase (SOD)		
	Aluminum oxide (Al_2_O_3_)	(19, 76)
	Copper (Cu)	(65, 77)
	Copper oxide (CuO)	(22, 66, 67)
	Copper sulfate (CuSO_4_)	(65, 77)
	CuInS2/ZnS quantum dots (QDs)	(78)
	Gold (Au)	(79–81)
	Iron oxide (Fe_3_O_4_)	(70)
	Silver (Ag)	(30, 34, 35, 72, 82)
	Silver nitrate (AgNO_3_)	(34)
	Single wallet carbon nanotubes (SWCNTs)	(37)
	Titanium dioxide (TiO_2_)	(39, 83)
	Zinc oxide (ZnO)	(19, 41, 73, 74)
Catalase (CAT)		
	Aluminum oxide (Al_2_O_3_)	(19, 76)
	Cobalt ferrite (CoFe_2_O_4_)	(56)
	Copper (Cu)	(65, 77)
	Copper oxide (CuO)	(22, 66, 67)
	Copper sulfate (CuSO_4_)	(65, 77)
	Gold (Au)	(80, 81)
	Iron (Fe)	(84)
	Iron oxide (Fe_3_O_4_)	(70)
	Silica coated iron oxide (Fe_3_O_4_-SiO_2_)	(59)
	Silver (Ag)	(30, 35, 82)
	Single wallet carbon nanotubes (SWCNTs)	(37)
	Titanium dioxide (TiO_2_)	(39, 83)
	Zinc oxide (ZnO)	(19, 41, 73, 74)
Glutathione peroxidase (GPx)		
	Copper oxide (CuO)	(66, 67)
	Gold (Au)	(80, 81)
	Silica coated iron oxide (Fe_3_O_4_-SiO_2_)	(59)
	Silver (Ag)	(35)
	Titanium dioxide (TiO_2_)	(39)
	Zinc Oxide (ZnO)	(73)
Glutathione sulfotranferase (GST)		
	Aluminum oxide (Al_2_O_3_)	(19, 76)
	Cobalt ferrite (CoFe_2_O_4_)	(56)
	Copper oxide (CuO)	(22, 67)
	Fullerene C_60_	(68)
	Gold (Au)	(80, 81)
	Iron oxide (Fe_3_O_4_)	(70)
	Silica coated iron oxide (Fe_3_O_4_-SiO_2_)	(59)
	Silver (Ag)	(34, 82)
	Silver nitrate (AgNO_3_)	(34)
	Titanium dioxide (TiO_2_)	(39)
	Zinc oxide (ZnO)	(19, 41)
Glutathione (GSH)		
	Aluminum oxide (Al_2_O_3_)	(76)
	Copper (Cu)	(65, 77)
	Copper oxide (CuO)	(67)
	Copper sulfate (CuSO_4_)	(65, 77)
	Fullerenes C_60_	(85)
	Gold (Au)	(81)
	Silver (Ag)	(72)
	Titanium dioxide (TiO_2_)	(39, 83)
	Gold (Au)	(17)
	Iron oxide (Fe_3_O_4_)	(70)
	Silica coated iron oxide (Fe_3_O_4_-SiO_2_)	(59)
Oxidized glutathione (GSSG)		
	Copper oxide (CuO)	(67)
**BIOMARKERS OF OXIDATIVE STRESS**		
Malondialdehyde (MDA)		
	Cobalt ferrite (CoFe_2_O_4_)	(56)
	Copper (Cu)	(65, 77)
	Copper oxide (CuO)	(24)
	Copper sulfate (CuSO_4_)	(65, 77)
	CuInS2 quantum dots (QDs)	(78)
	Silver (Ag)	(72)
	Titanium dioxide (TiO_2_)	(86)
	Zinc oxide (ZnO)	(41, 73, 74)
	ZnS quantum dots (QDs)	(78)
Lipid peroxidation (LPO)		
	Aluminum oxide (Al_2_O_3_)	(19, 83)
	Cobalt ferrite (CoFe_2_O_4_)	(56)
	Copper oxide (CuO)	(22, 66, 67)
	Fullerenes C_60_	(85)
	Silica coated iron oxide (Fe_3_O_4_-SiO_2_)	(29)
	Silver (Ag)	(30, 34, 35, 72, 87)
	Silver nitrate (AgNO_3_)	(34)
	Single wallet carbon nanotubes (SWCNTs)	(38)
	Titanium dioxide (TiO_2_)	(39, 83, 88)
	Zinc oxide (ZnO)	(19, 41, 74)
Myeloperoxidase activity (MPO)		
	Iron oxide (Fe_2_O_3_)	(27)
	Zinc oxide (ZnO)	(40)
Protein carbonyl (PC)		
	Copper oxide (CuO)	(66, 67)
Cytokines		
	Carbon Nanotubes (CNTs)	(89)
	Cerium oxide (CeO_2_)	(42)
	Copper (Cu)	(65, 77)
	Copper sulfate (CuSO_4_)	(65, 77)
	Gold (Au)	(80, 81)
	Hydroxylated fullerenes (C_60_(OH)_24_)	(44)
	Iron oxide (Fe_2_O_3_)	(42)
	Silver (Ag)	(60, 90)
	Titanium dioxide (TiO_2_)	(42, 48, 62, 91, 92)
	Zinc oxide (ZnO)	(42)
Lysozyme		
	Copper (Cu)	(77)
	Copper sulfate (CuSO_4_)	(77)
	Iron oxide (Fe_2_O_3_)	(27)
	Zinc oxide (ZnO)	(40)

**Table 2:** Species used in the evaluation of nanoparticles' effect on fish health.

**Species**	**Nanoparticles**	**References**
***Apistogramma agassizii***		
	Copper oxide (CuO)	(22)
***Anguilla anguilla***		
	Silica coated iron oxide (Fe_3_O_4_-SiO_2_)	(29, 59)
***Carassius auratus***		
	Aluminum oxide (Al_2_O_3_)	(19)
	Cerium oxide (CeO_2_)	(42)
	Iron oxide (Fe_2_O_3_)	(42)
	Titanium dioxide (TiO_2_)	(42)
	Zinc oxide (ZnO)	(19, 42)
***Catla catla***		
	Zinc oxide (ZnO)	(41)
***Chapalichthys pardalis***		
	Silver (Ag)	(35)
***Cyprinodon variegatus***		
	Copper oxide (CuO)	(24)
***Cyprinus carpio***		
	Zinc oxide (ZnO)	(93)
***Danio rerio***		
	Carboxylated polystyrene	(57)
	Cerium oxide (CeO_2_)	(75)
	Cobalt ferrite (CoFe_2_O_4_)	(56)
	Copper (Cu)	(20, 21)
	Copper oxide (CuO)	(66, 75, 94)
	Fullerene C_60_	(68, 95)
	Graphene oxide (GO)	(25)
	Gold (Au)	(57, 79, 96)
	Poly (lactic-co-glycolic acid) (PLGA)	(57)
	Polyaminoamine (PAMAM)	(97)
	Silver (Ag)	(21, 32, 60, 90, 98)
	Silica (Si)	(46, 47, 50)
	Single wallet carbon nanotubes (SWCNTs)	(37, 92)
	Titanium dioxide (TiO_2_)	(92, 95)
	Zinc oxide (ZnO)	(73, 74, 99)
***Dicentrarchus labrax***		
	Titanium dioxide (TiO_2_)	(62, 91)
***Epinephelus coioides***		
	Copper (Cu)	(65, 77)
	Copper sulfate (CuSO_4_)	(65, 77)
***Gobiocypris rarus***		
	CuInS_2_ quantum dots (CuInS_2_ QDs)	(78)
	ZnS quantum dots (ZnS QDs)	(78)
***Ictalurus punctatus***		
	Cobalt oxide (Co_3_O_4_)	(64)
	Copper oxide (CuO)	(64)
	Titanium dioxide (TiO_2_)	(64)
	Zinc oxide (ZnO)	(64)
***Labeo rohita***		
	Silver (Ag)	(30, 82)
***Micropterus salmoides***		
	Fullerenes C_60_	(85)
	Iron oxide (Fe_2_O_4_)	(100)
***Oncorhynchus mykiss***		
	Cadmium sulfate quantum dots (CdS QDs)	(54, 55)
	Cadmiun telluride quantum dots (Cd-Te QDs)	(51, 54, 55)
	Copper (Cu)	(43)
	Copper oxide (CuO)	(52)
	Gold (Au)	(43, 69)
	Carbon nanotubes (CNTs)	(89)
	Poly (acrylic acid)	(58)
	Silver (Ag)	(33, 34, 43, 69, 87, 101)
	Silver nitrate (AgNO_3_)	(34)
	Single wallet carbon nanotubes (SWCNTs)	(102)
	Titanium dioxide (TiO_2_)	(88)
***Oncorhynchus tshawytscha***		
	Aluminum oxide (Al_2_O_3_)	(76)
	Copper oxide (CuO)	(67)
	Titanium oxide (TiO_2_)	(83)
***Oreochromis niloticus***		
	Copper oxide (CuO)	(23)
	Iron oxide (Fe_2_O_3_)	(27)
	Multi walled carbon nanotubes (MWCNTs)	(28)
	Silver (Ag)	(61, 103)
	Zinc oxide (ZnO)	(40)
***Oryzias latipes***		
	Graphene– Titanium dioxide (GTiO_2_)	(104)
	Iron (Fe)	(70, 84)
	Silver (Au)	(72, 105)
	Titanium dioxide (TiO_2_)	(104)
***Paracheirodon axelrodi***		
	Copper oxide (CuO)	(22)
***Pimephales promelas***		
	Hydroxylated fullerenes (C_60_(OH)_24_)	(44)
	Polycarbonate	(45)
	Polystyrene	(45)
	Titanium dioxide (TiO_2_)	(48, 63)
***Poecilia reticulata***		
	Iron oxide (Fe_2_O_3_)	(49)
***Poeciliopsis lucida***		
	Polyamidoamine (PAMAM)	(71)
	Zinc oxide (ZnO)	(106)
***Prochilodus lineatus***		
	Titanium dioxide (TiO_2_)	(39)
***Scophthalmus maximus***		
	Silver (Ag)	(107)
***Sparus aurata***		
	Gold (Au)	(17, 26, 80, 81)
***Trachinotus carolinus***		
	Titanium dioxide (TiO_2_)	(53)

A biomarker can be any measurable biological response that reproducibly changes upon exposure to environmental pollutants (108). Recently, innate immunity has yielded a wide set of biomarkers for immunotoxicity against multiple xenobiotics including metal ions, pesticides, oil products, and chlorinated hydrocarbons (4, 9, 109–111). Among the most commonly used we find: leukocytes dynamics, phagocytic activity, lysozyme production, production of antimicrobial peptides, cytokines expression, and reactive oxygen species (ROS) production (110). Multiple studies have shown that innate immune parameters display high sensitivity to NPs. The conservation of innate parameters across animal models further makes them amenable for complementary studies, for instance, when toxicity to aquatic, and terrestrial ecosystems is of interest. Finally, early kinetics of innate parameters induction coupled to their sensitivity to capture additive or synergistic effects from environmental contaminants makes them a powerful alternative to evaluate eco-toxicity. In this review, we have drawn on recent literature to highlight those innate immunity biomarkers most often used in the assessment of teleost fish health exposed to NPs.

## External Barriers

External barriers to microbes infecting fish encompass mucous secretion produced in multiple tissues such as gills, skin, and intestine. These surfaces constitute physical barriers supported by immunologically-active agents such as lysozyme, complement, lectins, proteolytic enzymes, antimicrobial peptides, and reactive chemical species (112–116). While some of these defense molecules have been used as biomarkers in fish ecotoxicology (117), just a small proportion of these have been applied to the assessment of fish health after NPs exposure. Here, we discuss their use to date and how researchers can take advantage of them to assess NP toxicity.

### Mucus

The amount and biochemical content of mucus have been used as biomarkers for the evaluation of teleost fish health exposed to NPs. Mucus on the fish skin is a first line of defense, forming a barrier protecting tissues from the surrounding environment. This barrier is secreted by goblet cells and composed mainly of water, mucopolysaccharides, mucoproteins, and other soluble materials (114). Mucus, as part of the innate immune system, plays an essential role in protecting fish from xenobiotic exposure (114, 118). When fish health is compromised, the mucosal matrix may also be affected. Therefore, it can be a useful tool to assess the effect of NPs in fish. Nevertheless, only two studies have evaluated skin mucus after exposure to NPs (Table 1). In the first study, Oliveira et al. presented a non-invasive method to assess the effects of gold NPs (Au) in *Sparus aurata* by analyzing skin mucus (17). Measurements of total antioxidant capacity and esterase activity showed the sensitivity of skin mucus exposed to NPs, even at low concentrations (17). The second study in *Pimephales promelas* showed an increase in mucus production between 4 and 24 h after exposure to silver nitrate NPs (AgNO_3_) (18). By day 3, fish had considerably reduced their ability to produce mucus (18). The lack of additional studies employing mucus might be related to the limited knowledge concerning the repertoire of immune factors present in skin mucus and their precise protective role to study fish health fitness (118). Additionally, there is an inherent complexity in obtaining enough amount of mucus to analyze. Other potential biomarkers in mucus skin could be the quantification of activity of well-characterized enzymes such as protease and lysozyme (119). These prospective biomarkers may contribute to assess fish responses against a pathogen after NPs exposure. In addition, other immune-related parameters such as mucosal IgT secretion levels and the capacity for development of mucosal memory will allow the evaluation of fish health following NP exposure. These non-invasive biomarkers can be meaningful to study the fish health fitness while avoiding fish slaughter, thus, reducing the number of individuals used in environment pollution monitoring. Nevertheless, more studies are needed to define whether these parameters of skin mucus maintain consistent results in multiple experimental conditions such as NP type, concentration and exposure time.

### Gills

In fish, gills are the main organs for gas exchange and have a relevant role in ionic and osmoregulatory function (120). Moreover, gills are considered to be the most sensitive organ to a majority of xenobiotics because, like the mucus on the skin, gills are in direct contact with the environment (121). However, that may not apply to NPs. In gills, evaluation of NPs effects have been performed mainly by histopathology (19–23, 25, 28, 30–33, 39, 40), NP bioaccumulation (21, 23, 24, 27, 32, 34, 37, 39) and measurement of oxidative stress biomarkers (19, 22, 24, 25, 29, 30, 34, 35, 37, 39, 41) (refer to Oxidative stress section and Table 1). Regarding histopathology, some inaccurate histopathology-based results have been published due to the high degree of expertise necessary (122). For instance, controversial results have been published regarding the effects of silver NPs (Ag) in *Danio rerio*. Some studies report histopathological lesions in gills such as hyperplasia and inflammation after exposure of Ag NPs for 4 and 21 days at different concentrations (1.5–15 μg/L) (31, 36, 123). Other studies showed lack of pathology in gills after 28 days at Ag NPs concentrations between 10 μg/L and 1 mg/L (21, 32). These gill lesions would affect oxygen intake, osmoregulation, acid-base balance, and excretion of nitrogenous waste that in turn would likely produce acute toxicity (117). As a first line of contact between the host and its environment, one would expect gills to offer an optimal site to assess NP bioaccumulation. However, different studies showed that gills are not the main target for NP accumulation. For example, *Oreochromis niloticus* exposed to iron oxide NPs (Fe_2_O_3_) for 60 days showed greatest bioaccumulation in the spleen whereas gills displayed much lower levels of bioaccumulation (spleen > intestine > kidney > liver > gills > brain > and muscle) (27). Another study also using *O. niloticus* but exposed to copper NPs (Cu) for 30 days yielded a similar conclusion (bioaccumulation levels: liver > kidney > gills > skin > and muscle) (23). Bruneau et al. also described a higher bioaccumulation in liver than in gills in *O. mykiss* after an exposure of Ag NPs for 4 days (34). Thus, although NPs can be absorbed by the gills, they are not the preferred uptake route in teleosts (124). In general, histopathology, and NP bioaccumulation in gills might not be the best biomarker of NPs toxicity. They must be complemented with others potential biomarkers for NPs. For example, the number of goblet cells or mucus production are relevant biomarkers in aquatic toxicology that can be tested to evaluate NPs effects on fish health (117).

## Cellular Response

### Leukocytes

Immune cells play a pivotal role in the clearance of pathogens or other foreign elements like NPs (125). Thus, assessment of leukocyte engagement may provide insights into NP toxicity. Indeed, kidney NP bioaccumulation is linked to a reduction in neutrophils function and a possible reduction in their ability to control bacterial infections (48). In this section, we review recent reports on the effects of NPs on the dynamics and functionality of fish leukocytes (also summarized in Table 1). Additional effects on leukocyte responses will be addressed in the following sections (refer to Internalization of nanoparticles, Oxidative stress, and Cytokines sections).

### Macrophages

Resident macrophages offer early detection of insults (e.g., pathogen infiltration, tissue damage, toxicant exposure) at various tissue sites (126, 127). Changes of macrophage viability and function have long been implicated with toxicity resulting from exogenous pollutants (e.g., metals, sewage, hormones disrupting compounds, pharmaceuticals chemicals) (12, 110, 128). Thus, macrophages and their proper function can be used as biomarkers for immunotoxicity (12). Most recently, this modulation in macrophage function has been used as biomarkers to evaluate NPs toxicity (Table 1). For instance, TiO_2_ NPs induced upregulation of macrophage colony-stimulating factor 1 (MCSF-1) in multiple pooled tissues of *P. promelas* (anterior kidney, liver, spleen and gills) (48). Changes in MCSF-1 gene expression have been associated with impairment of macrophage function. This is consistent with its important role in viability, differentiation, mobilization, and activation of macrophages and their precursors (48). Using an *in vitro* approach, the effect of carbon nanotubes on kidney mix population of macrophages in *O. mykiss* revealed that SWCNT induces upregulation of pro-inflammatory cytokine expression such as IL-1β but not in IFNα expression, indicating a selective pathway (89). These results suggest a different effect in pro-inflammatory soluble mediators. Duan et al. evaluated the effect of sublethal increasing concentrations of silica NPs (Si) on macrophage function using zebrafish embryos (50). Results showed a downregulation of gene expression in macrophage inhibitory factor (MIF) and vascular endothelial growth factor receptor 2 (VEGFR2). The higher Si NP concentration, the stronger the down regulatory effect resulting in a decrease in macrophage activity (50). These results showed that even at sublethal doses of NPs, macrophage gene expression may provide useful read-outs for evaluation of fish immunity. Melanomacrophages are highly pigmented macrophage type that possess phagocytic function and play an important role in the immune response (129). Melanomacrophage centers (MMCs) have been used as biomarkers to assess fish health and aquatic environmental pollution (130–132). In this context, using the *P. reticulata* model, it has been shown that citrate-functionalized maghemite (γ-Fe_2_O_3_) induces significant changes in MMCs in liver. Acute (3–7 days) and chronic exposure (14–21 days) to γ-Fe_2_O_3_ NPs increase number, cellular content and size in MMCs, suggesting that γ- Fe_2_O_3_ might be involved in the regulation of innate immune responses (49). Altogether, these demonstrates that NPs are capable of interfering with multiple aspect of macrophage function (4). Due to the limited number of observations on the responses of MCSF-1, gene expression of pro-inflammatory cytokines, and immune-related genes, and MMCs, these parameters cannot yet be considered as valid biomarkers for NPs toxicity purposes. However, the key role of these parameters in macrophage function motivates continued research on its feasibility as a biomarker.

### Neutrophils

Neutrophils are polymorphonuclear leukocytes that are central to the induction and regulation of acute inflammation (133, 134). Neutrophils act as the predominant phagocytic cells for first-line defense to be recruited to an inflammatory site against diverse xenobiotics (135, 136). Thus, neutrophils play an important role in recognizing and eliminating foreign agents, including some NPs (137). In this regard, NPs have been shown to not only impair neutrophil functionality but also affect a diverse array of biochemical responses. These effect in neutrophils features have been used as biomarkers to evaluated NPs toxicity (Table 1). However, based on these studies, there is little clarity to date on the effects of NPs on neutrophil biology. For example, fullerenes have shown to inhibits some function such as ROS production and NETosis in mixed neutrophil populations from kidney of *P. promelas* (44). Interestingly, this effect did not induce significant changes in total neutrophil counts, suggesting that neutrophil antimicrobial functions are the primarily affected following fullerene internalization rather than neutrophil development *per se* (44). On the other hand, Jovanovic et al. revealed that TiO_2_ NPs injections in *D. rerio* increased neutrophil migration, oxidative burst and phagocytic activity, associated to higher tissue damage (48, 95). In this context, increased neutrophil activation can be associated to phosphorylation of p38 mitogen-activated protein kinase (MAPK) and extracellular signal-regulated kinases. Conversely, TiO_2_ NPs downregulated matrix metalloproteinase 9 (neutrophil elastase) expression, which further decreased IL-8 neutrophil mobilization from hematopoietic tissues (95). Małaczewska and Siwicki et al. found that even when Ag NPs did not induce an effect on peripheral neutrophils, spleen phagocytic activity was inhibited only at high concentrations of Ag NPs using the *O. mykiss* model (43). These results might be attributed to differences in concentration and time of exposure (43). Ortega et al. used PAA functionalized NPs [cerium oxide (CeO_2_), TiO_2_, Fe_2_O_3_ and zinc oxide (ZnO)] to evaluate their effect on function of a mixed neutrophil population of kidney in *Carassius auratus* (42). Here, it was shown that NPs decreased neutrophil viability in a concentration and time-dependent manner. Additionally, lower concentration of NPs decreased neutrophil degranulation, and increased ROS production along with an increase of gene expression of pro- and anti-inflammatory cytokines in neutrophils such as IL-1β and IFNα. Hence, these sub-lethal doses of NPs might be linked to a higher susceptibility against pathogens and might thus impair fish health (42). Altogether, these studies reveal that several neutrophil functions can be modulated following NPs exposure. However, depending upon the type of NPs evaluated, different results can be observed. Based on the small number of studies to date, it is difficult to estimate the reproducibility of these findings and the reliability of each neutrophil function as biomarkers. Nevertheless, any disruption in neutrophil function will represent major implication of fish immune defenses. Therefore, more studies are needed to clarify if these neutrophil parameters could be used as biomarker to monitor NPs toxicity in fish.

### Lymphocytes

Lymphocytes offer a diverse array of effector and regulatory functions in multiple tissues. They are typically associated to long-term immune responses; however, a wide group of lymphocytes has relevance in innate defenses. For instance, phagocytic B lymphocytes participate in initial responses following pathogen invasion (138). Furthermore, innate lymphoid cells (ILCs) like cells, a recently described group in zebrafish (139), have a role in inflammation, tissue remodeling, and homeostasis regulation (140, 141). Thus, lymphocyte development, viability and proper function have a novel potential as biomarker for NP toxicity. For example, *O. mykiss* exposed to Ag NPs, Au NPs and Cu NPs revealed a differential effect on lymphocytes isolated from blood and spleen by density gradient separation. Lymphocyte viability was decreased only by Ag NPs following two-day incubation. However, no effect on lymphocyte proliferation was observed at the same concentration while enhanced proliferation rates were stimulated with lower concentration of Ag NPs. Conversely, Au NPs had the strongest effect on lymphocyte proliferation within the NPs tested (43). In addition to the effect of NPs on lymphocyte viability and proliferation, there are descriptions of interaction with other pollutants that potentiate their overall effect. For example, *in vivo* exposure of *Dicentrarchus labrax* to TiO_2_ NPs, showed a cytotoxic effect when it interacted with dioxins in splenocytes, suggesting that the spleen might be a possible biomarker for NP toxicity due to NP bioaccumulation in this tissue (53, 91). These changes in lymphocyte development will further promote changes in the overall total lymphocyte counts. This was shown by Khabbazi et al. who found significant differences in lymphocytes counts in blood in *O. mykiss* exposed to copper oxide NPs (CuO) (52). On the other hand, *P. promelas* exposed to hydroxylated fullerenes or TiO_2_ NPs did not produce differences in lymphocytes counts in blood samples (44, 48). Due to the limited number of studies and notable difference between results in viability and lymphocyte counts after an exposure with NPs, these parameters cannot yet be considered as biomarkers to assess NPs toxicity. Although most studies discussed here used morphological approaches for lymphocyte definition, further studies are needed for deeper and more accurate characterization of lymphocyte subsets in fish and their specific contribution in NP toxicity.

### Internalization of Nanoparticles

Internalization of invading pathogens or threats is the paramount to immune defense in several organisms. In the case of NP, their size, shape, surface chemistry, and mechanical properties influences the mechanism of cellular internalization (142, 143). For example, following entry, small NPs (< 100 nm) are rapidly internalized by professional cells through endocytosis whereas large NPs (larger than 500 nm) are uptaken by micropinocytosis or phagocytosis (34, 144). Thus, despite their origin and internalization processes, NPs have been shown to modulate cellular responses in a variety of professional phagocytes such as neutrophils, monocyte, and macrophages (43). For instance, Malaczewska and Siwicki evaluated the effect of Ag NPs, Au NPs and Cu NPs on the phagocytic activity of spleen leukocytes from *O. mykiss*, showing an increase in phagocytic capacity in fish exposed to Ag NPs, but not observed in animals exposed to Au NPs nor Cu NPs (43). Through intraperitoneal injections, TiO_2_ NPs were administrated to *Trachinotus carolinus* and cellular uptake was evaluated after 3 days. Results demonstrated NP uptake in the liver, kidney, lung, and spleen following a similar process that is also observed in mice and human cells (145, 146). However, the authors suggested that other internalization processes might occur in fish other than phagocytosis since they detected TiO_2_ NPs reaching the nucleus compartment. Bruneau et al. have shown that cadmium tellurium quantum dots (Cd-TeQDs) induce an increase in phagocytosis rates associated to the size and form of those NPs in *O. mykiss* (51). In a different experiment, Greven et al. demonstrated that aggregation of polystyrene and polycarbonate nanoplastic particles promotes NPs internalization in *P. promelas* (45). As described, NP induced significant changes in the capacity of leukocytes to perform internalization of NPs. This suggests a regulatory effect, occurring in a time, and concentration-dependent manner, that is associated to the type of NP. Studies summarized here suggest that internalization of NPs by phagocytic cells, is a potential biomarker that can offer relevant information about the effect of the NPs at the intracellular level and should be complemented with other biomarkers to gain an integrative view of altered cellular function.

### Oxidative Stress

Once NPs have gained access to the organism, distributed to multiple tissues, and internalized into cells, they are capable to promote intracellular responses such as oxidative stress. Oxidative stress refers to the imbalance between the production of free radicals and the protective antioxidant defense system (109, 147). Oxidative stress has become a relevant biomarker for aquatic toxicology (148), because this phenomenon in fish can be triggered by many chemicals including NPs, metal ions, pesticides, oil products, and chlorinated hydrocarbons (9, 95, 109–111). These environmental pollutants can induce oxidative stress in fish via two ways: directly by affecting the animals or indirectly by modifying the environmental conditions (111). Oxidative stress can be detected and measured giving a quantitative indication of fish health status and it can be evaluated using different parameters such as free radicals, antioxidant defenses and biomarkers of oxidative stress (Table 1).

### Reactive Oxygen Species and Nitric Oxide

ROS are free radicals formed as a natural by-product of the normal metabolism of oxygen. ROS have important roles in cell signaling and homeostasis (149). However, ROS may significantly increase under stressful scenarios such as infection, inflammation, and exposure to environmental pollutants (111, 150). This increase may disrupt homeostasis, producing damage at the cellular level, and disease or death at the organism level (109, 148). ROS has been one of the most common parameters used for the evaluation of fish health after exposure to different types of NPs (Table 1). The preference of ROS as a biomarker is attributed to: (i) its high sensitivity to different NP exposure; (ii) fast-triggered response compared to other free radicals; (iii) remarkable consistency of results between studies; and (iv) increasing development of novel highly-sensitive reagents that allow the detection of slight changes of ROS production. NPs that have been shown to induce ROS production in fish include carbon-based, metal-based, plastic-based, and polymeric carrier NPs.

Levels of ROS have been successfully measured after exposure to NPs in different fish species such as *D. rerio* (25, 37, 46, 56, 60, 66, 68, 73, 74, 94), *P. promelas* (44, 45, 48), *Oryzias latipes* (70, 72), *Poeciliopsis lucida* (71, 106), *O. niloticus* (27, 40), *C. auratus* (42), *O. mykiss* (43), *Oncorhynchus tshawytscha* (67), *Epinephelus coioides* (65), *Ictalurus punctatus* (64), *Anguilla anguilla* (29), *Apistogramma agassizii* (22), *Paracheirodon axelrodi* (22), and *Prochilodus lineatus* (39). Interestingly, these studies showed a high versatility of ROS that can be measured in different stage of fish development, tissues, and cells. Most studies using embryos (46, 47, 56, 66, 73, 74) and larvae (70) observed an increase of ROS levels. However, an exception were those studies using Si NPs where no effect was observed in embryos (46, 47). These authors claimed that the lack of increase in ROS levels was related to the type of NP used (47). The use of embryo/larvae models to analyze ROS levels confer an integrative view of whole organism response. Even when some studies showed that no effect was observed in ROS levels, use of embryos/larvae confer a low cost and easy handling tool that still make them valuable as biomarkers. In the case of adult fish exposed to NPs, there are studies that assess ROS production in whole organs. ROS have been measured in different tissues such as gills (22, 25, 37, 39), liver (37), intestine (37), and brain (37), and fluids as serum (27, 40). Using gills, different studies showed a persistent ROS activation after NP exposure. For instance, *A. agassizii* exposed to Cu NPs showed greatest increase in ROS production at 3 days of exposure (22). Souza Khabbazi et al. observed a significant increase in ROS levels at day 1 of exposure with graphene oxide (GO) in gills of zebrafish (25). As first line of contact between the host and NPs, one would expect gills to offer a faster biomarker than internal organs. However, a study also using zebrafish but exposed to SWCNT showed a significant increase in ROS production in gills at 3 days of exposure comparatively slower than in liver and brain, where this increase was at 2 days (37). No modulatory effect was observed in the intestine (37). The available information reveals that measurement of ROS in gills is a valid biomarker to assess NPs toxicity, even though time of response can be slower than other organs. Furthermore, ROS production have been evaluated *in vitro* using both cell lines and primary cell cultures. These studies took advantage of a large variety of cell types such as: zebrafish liver (ZFL) (60, 68, 94); PLHC-1 derived from hepatocellular carcinoma in *P. lucida* (71, 106); and CHSE-214 derived from *O. tshawytscha* embryo (67); and primary cell cultures such as: neutrophils (42, 44, 45, 48); hepatocytes (64, 65, 69); splenocytes (43) and phagocytes (29). In cell lines, different NPs, concentrations, and exposure time, increased ROS levels. In contrast, not all NPs successfully modulate ROS production in primary cell cultures (42, 69). These results reveal that ROS production in cell lines, as per their homogeneous nature, is more consistent than in primary cell-based assays. This might be attributed to factors such as heterogeneity, cellular viability, and activation state of primary cells. Diverse studies have been done on a wide range of fish species and targets that can be used to test NPs effect on ROS production. These studies point out ROS as a reliable, sensitive, and valid biomarker of NPs toxicity in fish.

NO is the second free radical used as a biomarker to assess NPs toxic effects in fish described in literature. NO is produced by inducible nitric oxide synthase (iNOS). iNOS converts L-arginine and oxygen into L-citrulline and NO (151). NO may produce a wide range of physiological and pathophysiological effects (152). In contrast to ROS, there are only two studies which have evaluated NO expression in fish after exposure to NPs (Table 1). Based on these studies, it appears that NO may also serve as a useful biomarker. In the first study, zebrafish embryos exposed to CuO NPs showed a significant increase in NO production. Interestingly, the same increasing trends were observed for ROS production (66). In a second study, also using zebrafish embryos two NPs were analyzed, CuO NPs, and CeO_2_ NPs. CuO NPs increased NO levels in contrast to CeO_2_ NPs that decreased them (75). The authors explained that this difference was due to the high toxicity of CuO and the ability of Ce to scavenge NO (75). The low number of studies that focus on NO production may be due to the response rate of this free radical, where ROS levels have been shown to respond faster than NO. As mentioned above, NO levels need to be produced by iNOS expression and this specific pathway is slower than ROS production (153). As showed above, since only two studies analyze the effect on NO more research is required to determine its potential use as a biomarker in the context of NPs toxicity. Among other, evaluation of diverse NPs at different concentrations that can confirm that NO reproducibility of the results.

### Antioxidant Defenses

Free radical levels can be balanced by the antioxidant system which scavenges free radicals and delays or inhibits cellular damage (154, 155). There is a wide range of antioxidant defenses as diverse as free radicals themselves (156). Antioxidant defenses have been widely used as biomarkers of environmental toxicology (11, 109). Different antioxidants have been analyzed as tools to measure the effects of NPs on fish health (Table 1). The three main antioxidants and direct free radical scavengers include superoxide dismutase (SOD), catalase (CAT), and glutathione peroxidase (GPx). Other antioxidant enzymes that inactivate secondary metabolites have been measured to evaluate the effects of NPs on fish health such as glutathione sulfotransferase (GST), total glutathione (GSH), glutathione in its reduced state (GR), and its oxidized state (GSSG) (157, 158) (Table 1). Antioxidant levels have been measured mainly by biochemical assays but also by gene expression studies (65, 70, 79–81). As a homeostasis system, we would expect that antioxidants defenses levels increase after NPs exposure. However, some studies showed that antioxidants defenses do not always showed these patterns. For instance, Srikanth et al. used chinook salmon cells (CHSE-214) exposed to CuO NPs, they found a significant increase in ROS, SOD, CAT and GPx levels as expected (67). In contrast, Ganesan et al. observed a marked decrease in antioxidant defenses (SOD, CAT and GPx) in zebrafish embryos after exposure to CuO NPs, however, ROS levels showed significant increases (66). The authors claimed that the antioxidant system was overpowered by increased ROS production (66). Despite that multiple studies have used only antioxidant defenses to assess fish health following NPs exposure (30, 34, 41, 59, 76, 77, 79–83), we point out that antioxidant defenses should be measured as a complement of ROS and not by themselves.

### Biomarkers of Oxidative Stress

NPs are potent triggers of oxidative stress in fish and this can be detected through the measurement of molecular biomarkers. However, no single biomarker has been identified as sensitive and specific enough to detect oxidative stress alone (159). Generally, products of cells with oxidative stress or tissue damage are observed after exposure to NPs. Examples of these are malondialdehyde (MDA), lipid peroxidation (LPO), myeloperoxidase activity (MPO) and protein carbonyl (PC) (Table 1). As expected, these biomarkers display similar patterns of ROS production. For example, zebrafish larvae exposed to ZnO NPs showed a significant increase in ROS and MDA levels at different concentrations (73). Ganesan et al. reported similar results: an increase in ROS, NO, and biomarkers such as LPO and PC in zebrafish embryos after exposure to CuO NPs (66). Using an *in vivo* approach with ZnO NPs, plasma in *O. niloticus* revealed that both small and large NPs incremented both ROS and MPO levels, showing a clear activation of oxidative stress (40). Biomarkers of oxidative stress have lower specificity for NP toxicity (159), thus, they should be used to complement ROS and antioxidant defenses results following NP exposure.

### Cytokines

Cytokines are small proteins produced by immune cells that act as signaling molecules within the immune system (160). Thus, cytokines regulate inflammatory signals against pathogens or any external agent such as NPs. This modulation of cytokine expression has been used to assess fish health following NPs exposure and mainly through molecular assays such as qPCR (Table 1). The rising number of studies evaluating cytokine gene expression in NPs toxicity context is likely related to the increasing cost effectiveness and availability of molecular techniques such as qPCR, and the rapid progress in the sequencing of fish cytokines (161–164). In this regard, exposure of *S. aurata* to Au NPs causes altered gene expression in head kidney (80), when pro-inflammatory cytokines such as IL-1β and TNFα were upregulated after 4 days exposure. Interestingly, through the same technique, authors observed an increase in oxidative stress in head kidney (80). Picchietti et al. observed an increase in IL-8 and TGF-β expression, and internalization of TiO_2_ NPs in DLEC cells (a cell line established from *D. labrax*) after 1 day of exposure (62). Another study observed upregulated expression of IL-1β and TNF-α in intestine of *E. coioides* after exposure to Cu NPs for 25 days (77). Together, these results showed that cytokine gene expression is sensitive to NP exposure. Hence, cytokines emerge as a possible biomarker for monitoring the impact of NPs on overall fish health, since current methodologies provide sensitive platforms to assess variations at the molecular levels.

### Lysozyme

Lysozyme is a relevant defense component of the innate immune system through its antibacterial activity. Furthermore, lysozyme can also act as an opsonin and activate complement system and phagocytes (165). It is widely distributed in mucus, plasma, kidney, spleen, intestine, and gills (166–168). Lysozyme activity in plasma or serum is a standard ecotoxicological biomarker in fish (169). However, to date there are only three studies which have evaluated lysozyme activity in fish after exposure to NPs (Table 1). For example, *E. coioides* exposed to Cu NPs and copper sulfate NPs (CuSO_4_) revealed diminished lysozyme activity in the intestine after 25 days (77). This suppressive effect was also observed in blood samples in *O. niloticus* exposed to Fe_2_O_3_ NPs after 60 days (27). In another study, this decrease in lysozyme activity in the serum was only observed for large ZnO NPs at the lowest concentration on day 14 (40). Some studies have described that lysozyme are able to bind metal oxide NPs (170, 171) producing a decrease of lysozyme activity (171). Thus, NPs have a suppressor effect on lysozyme activity in fish. Hence, lysozyme activity represents a good indicator of immunosuppression that NPs can cause in fish. However, more research is needed to show the effectiveness of lysozyme as a biomarker due to the limited number of existing studies until today. Among others, analysis using different species, tissues (e.g., mucus on the skin), NPs and concentration of NP concurrently are needed to support consistent results. Ideally, performing a pathogen challenge after NP exposure would be best to assess the complete effect of NP-mediated lysozyme suppression on fish response to pathogen exposure.

## Industrial Point Of View

The industrial production of engineered NPs has grown at a considerable rate with an increasing number of commercial products utilizing them, which includes; paints, fabrics, cosmetics, treated wood, electronics, and sunscreen (172). Established in 2005, the Nanotechnology Consumer Products Inventory (CPI) listed 54 consumer products containing nanomaterials. Over 1,800 products from 622 companies in 32 countries are currently inventoried (173).

The rate of development for environmental exposure limits and monitoring policies has been surpassed by the growth rate of this emerging class of pollutants. The resulting gap in regulations for engineered NP has been identified as a key focus area in Europe and the United States through the European Commission and the National Institute for Occupational Safety and Health, respectively (174). Monitoring methodology able to detect NPs present in the environment is very limited, combined with inherent challenges for sampling, creating barriers in the ability to distinguish adverse effects (175).

To complement biological read-outs as those described above, analytical techniques used to measure regulated compounds typically rely on reactive potential within a closed system (i.e., conductivity, polarity, bond with coloring agent) thus allowing for cost effective high throughput production of samples. Required detection limits for NP's reduces options to highly sensitive instrumentation i.e., field-flow fractionation, size-exclusion chromatography, liquid chromatography, transmission electron microscopy (TEM), and atomic force microscopy (AFM) (176). Cost restrictions and equipment infrastructure are limitations preventing methodology viability.

Alternative methods based on bioanalytics are not bound by individual chemical structures, which enables them to assess the net effect on whole biological systems (177). The European Environment Agency (EEA) and the Australian Governments National Water Initiative (AU NWI) have directed significant resources to the evaluation of biology-based monitoring tools to ensure water quality. The European Union Water Framework Directive (EU WFD) and the Australian Governments National Urban Water and Desalination Plan have both found that bioanalytics offered a distinct advantage as a monitoring tool offering the only read-out that integrates the effects of complex mixtures during evaluation of water quality on important ecosystem functioning (177). Interactions of NPs with the innate immune system of fish elicit a number of several, quantifiable, and reproducible responses such as ROS, antioxidants defenses, internalization of NPs, and cytokine production. Therefore, biomarkers based on innate immune responses offers significant opportunities for the development of robust methodologies that can provide functional biological outputs assessing the health of an aquatic ecosystem following exposure to a variety of NPs.

## Concluding Remarks

**Figure 1:**
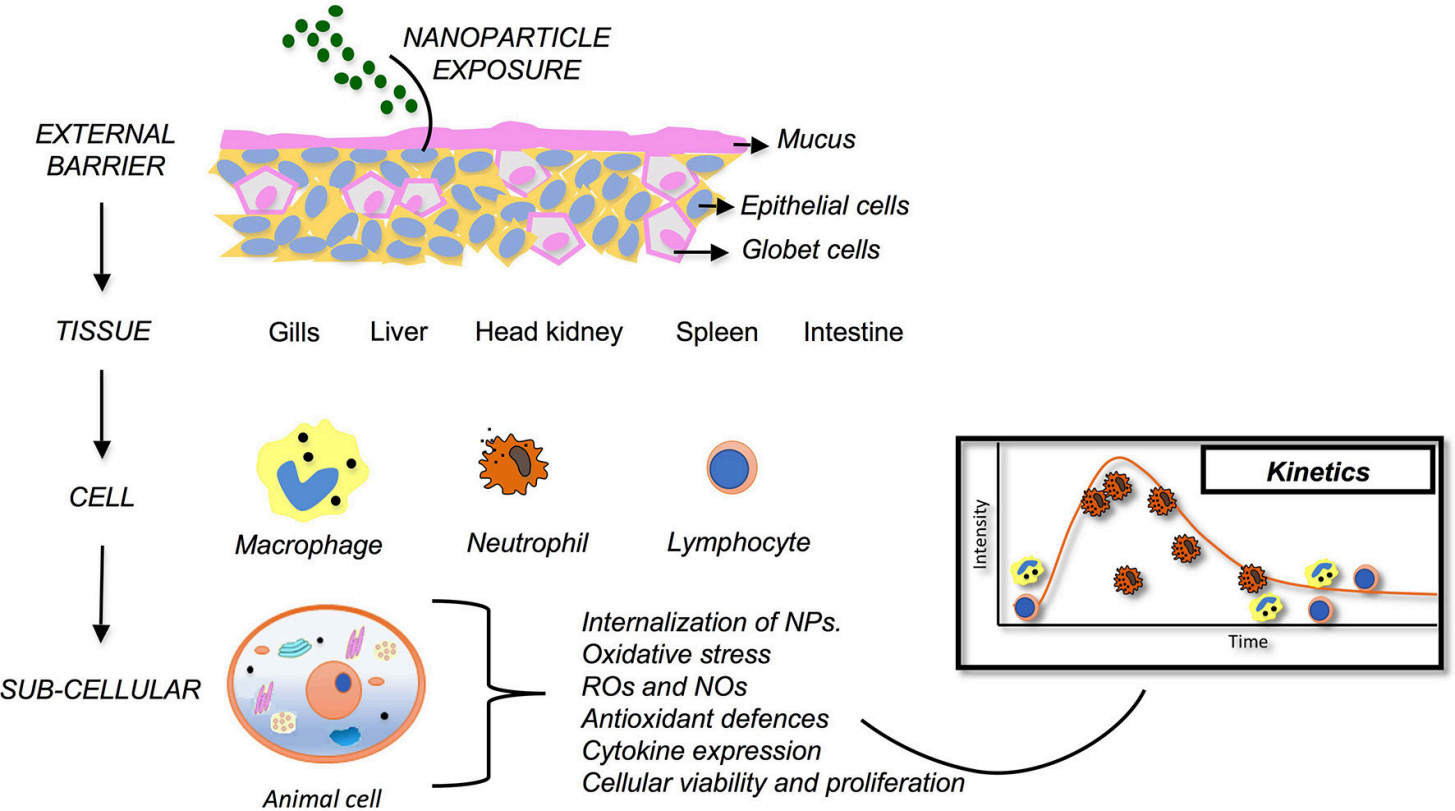
Innate immunity as a bioindicator of health for teleost fish exposed to nanoparticles. Following NP exposure, fish immunity is evident at different levels (external barriers, tissue, cellular and sub-cellular). Each provides unique insights into changes to homeostasis and, thus, can be used to detect nanoparticle-induced immunotoxicity.

The literature summarized here explores a range of possible innate immune biomarkers as tools for the assessment of fish health following NP exposure. These biomarkers reveal multiple alterations produced by NPs into diverse organs and tissues in multiple fish species (Figure 1). To date, oxidative stress is the most widely innate immune biomarker studied to demonstrate NPs toxicity in fish, particularly using the measurement of ROS, and antioxidant defenses. This is attributed to its high sensitivity to NPs exposure and the remarkable consistency of results among studies on a wide range of fish species and targets. These studies pointed out ROS and antioxidants defenses as a reliable, sensitive, and valid biomarkers of NPs toxicity in fish. Additionally, this review reveals other potential biomarkers that can be used to evaluated fish health following NPs exposure. For example, analysis of mucus on the skin, leukocyte functionality (macrophages and neutrophils), internalization of NPs, cytokine expression, and lysozyme levels. These potential biomarkers showed significantly promising results, although, more research is needed to determine their consistency and reliability. Furthermore, some innate immune parameters used on current literature are not valid options as biomarkers to evaluated NPs toxicity. For example, histopathology, and NP bioaccumulation in gills and biomarkers of oxidative stress. These parameters do not provide accurate and relevant information in NPs toxicity context. Despite these considerations, industry needs biomarkers as tracking tools to evaluate NPs toxicity, because analytical techniques present cost restrictions and equipment infrastructure limitations that constraint methodologies viability. In conclusion, it can be stated that innate immune biomarkers are promising tools to assess fish health following NPs exposure. However, much work must be done in order to test and interpret some of biomarkers responses present in this review. Based on the diversity of these parameters, the number of studies analyzing NP effects in fish is yet limited. Further studies on the impact of NPs will provide a better understanding of detrimental effects of NPs to fish health.

## Author Contributions

The conceptualization of the review was performed by DT and DB. DT was the primary writing contributor to this review. JM-B and JW wrote and edited parts of the manuscript. DT and DB edited the final manuscript. All authors approved the final version of the manuscript.

### Conflict of Interest Statement

The authors declare that the research was conducted in the absence of any commercial or financial relationships that could be construed as a potential conflict of interest.
